# Vitamin D deficiency and dyslipidemia in early pregnancy

**DOI:** 10.1186/s12884-015-0751-5

**Published:** 2015-11-26

**Authors:** Abdulrahman Al-Ajlan, Soundararajan Krishnaswamy, Majed S. Alokail, Naji J. Aljohani, Amal Al-Serehi, Eman Sheshah, Naemah M. Alshingetti, Mona Fouda, Iqbal Z. Turkistani, Nasser M. Al-Daghri

**Affiliations:** Department of Clinical Laboratory Sciences, College of Applied Medical Sciences, King Saud University, PO Box 10219, Riyadh, 11433 Kingdom of Saudi Arabia; Prince Mutaib Chair for Biomarkers of Osteoporosis, Biochemistry Department, College of Science, King Saud University, PO Box, 2455, Riyadh, 11451 Kingdom of Saudi Arabia; Specialized Diabetes and Endocrine Center, King Fahad Medical City, Faculty of Medicine, King Saud bin Abdulaziz University for Health Sciences, Riyadh, 11525 Saudi Arabia; Maternal-Fetal Medicine Department, King Fahad Medical City, Riyadh, 59406 Saudi Arabia; Diabetes Care Center, King Salman Bin Abdulaziz Hospital, Riyadh, Saudi Arabia; Obstetrics and Gynecology Department, King Salman Bin Abdulaziz Hospital, Riyadh, Saudi Arabia; Department of Medicine, Endocrinology Division, College of Medicine, King Saud University, Riyadh, 12372 Saudi Arabia; Department of Obstetrics and Gynecology, College of Medicine, King Saud University, Riyadh, 12372 Saudi Arabia

**Keywords:** 25(OH)D, Atherogenic, Cholesterol, Calcium, Hypovitaminosis D, Hypercholesterolemia, Hypertriglyceridemia

## Abstract

**Background:**

Vitamin D deficiency is a common nutritional issue and dietary supplementation in the general population, including pregnant women, is generally advised. Appropriately high levels of vitamin D are expected to play a role in containing the glycemic and atherogenic profiles observed in pregnancy. However, the relation between vitamin D status and the lipid metabolic profile in Saudi women, who are known to suffer from chronic vitamin D deficiency and high incidence of obesity and type II DM, during the course of pregnancy is not known.

**Methods:**

In this study, we analyzed the relation between serum vitamin D level and various serum metabolic markers among Saudi women (*n* = 515) in their first trimester of pregnancy (11.2 ± 3.4 weeks). Coefficients of Pearson correlation and Spearman rank correlation were calculated for Gaussian and non-Gaussian variables, respectively. Serum vitamin D status was defined as (in nmol/L): deficient (<25), insufficient (25–50); sufficient (50–75) and desirable (>75).

**Results:**

Results indicated that vitamin D status was sufficient in only 3.5 % of the study participants and insufficient and deficient in 26.2 % and 68.0 % of participants, respectively. Serum vitamin D values in the overall study population correlated positively with serum levels of total cholesterol (R = 0.172; *p* < 0.01), triglycerides (R = 0.184; *p* < 0.01) and corrected calcium (R = 0.141; *p* < 0.05). In the subgroup of vitamin D deficient subjects (*n* = 350), log serum vitamin D values correlated with serum triglycerides (R = 0.23; *p* = 0.002) and cholesterol (R = 0.26; *p* = 0.001).

**Conclusions:**

The positive correlations between serum vitamin D and the atherogenic factors such as total cholesterol and triglycerides indicate a pro-atherogenic metabolic status in vitamin D deficient expectant mothers. This may represent an adaptation to the high metabolic demands of pregnancy.

## Background

Hypovitaminosis D in pregnant women is very common and has important implications for the mother and lifelong health of the child, as it has been linked to maternal and child infections, small-for-gestational age (SGA), preterm delivery, preeclampsia, gestational diabetes mellitus (GDM), as well as DNA imprinting in the infant for lifelong chronic diseases [1].

Vitamin D deficiency is a worldwide public health problem in all age groups [2]. Globally, prevalence of deficient and insufficient vitamin D status ranges from 5 to 84 % in pregnant women [3]. Countries in the Middle-east, despite abundant sunshine, have reported lower mean vitamin D levels than others, and higher deficiencies in females than males have been attributed to their conservative clothing and time spent indoors [4, 5]. Because of the possibility of balancing vitamin D status through dietary supplementation, intense research is being pursued to gain an understanding of the various factors that affect vitamin D status and the effect of vitamin D deficiency on normal health.

Accumulating evidence links vitamin D deficiency with abnormal glucose and lipid metabolism, and epidemiologic studies have shown that women who develop GDM are more likely to be vitamin D deficient [6–8]. The three essential lipid indices (total cholesterol, triglycerides and LDL cholesterol) increase during pregnancy and thus mimic the markers of metabolic syndrome [9–11]. It was suggested that changes in carbohydrate and lipid metabolic markers in pregnant women may not reflect a pathologic condition but rather indicate a necessary adaptation of the mother’s physiology to balance the energy demands of the fetus and to prepare the mother for delivery and lactation [12]. In contrast, GDM was suggested to be a transient manifestation of long-standing metabolic dysfunction [10].

Even though *in vitro* and *in vivo* animal studies show a plethora of pathways regulated by Vitamin D, its role during fetal development is not well understood. Observational evidence links low maternal vitamin D status with an increased risk of non-bone health outcomes in the mother [13], but the totality of the evidence for health outcomes is oftentimes contradictory, lacks mechanistic explanations, and is inconclusive [14]. In the light of widespread occurrence of vitamin D deficiency, regulatory agencies make recommendations for significant dietary supplementation of vitamin D. However, uncertainty still exists regarding the optimum level of vitamin D in pregnant women relative to both maternal and fetal adverse health outcomes. In this respect, vitamin D status data from different populations are expected to aid in finding optimum levels. This study was performed to assess the vitamin D status in a population of pregnant Saudi women and to analyze its relation with various anthropometric and biochemical parameters, especially carbohydrate and lipid metabolic markers.

## Methods

### Subjects

A total of 515 hundred Saudi women in their 1^st^ trimester of pregnancy (11.2 ± 2.75^th^ week) were recruited from King Fahad Medical City (*n* = 134), King Khalid Hospital (288) and King Salman Hospital (93) in Riyadh, Saudi Arabia. Only Saudi women ages 18–35 years with previous clinical data regarding maternal characteristics, glycemic data and neonatal parameters were included in the study. Women with previous history of diabetes mellitus (type I or II), malignancy, and history of chronic kidney or liver disease were excluded. Written informed consent was taken from each subject before study inclusion. Approval was granted by the Ethics Committee of the College of Science, King Saud University, Riyadh, Kingdom of Saudi Arabia (KSA). Study subjects were asked to complete a general questionnaire containing demographic information, including past and present medical history, and to return after fasting for more than 10 h for anthropometry and blood withdrawal.

### Anthropometry and blood collection

Subjects were requested to visit their respective hospitals between 8:00 and 9:00 h following an overnight fast (>10 h) for anthropometry and blood withdrawal by the nurse and physician on duty, respectively. Anthropometry included height (rounded to the nearest 0.5 cm), weight (rounded to the nearest 0.1 kg), waist and hip circumference (centimeters), and mean systolic and diastolic blood pressure (mmHg, average of two readings). Body mass index (BMI) was calculated as weight in kilograms divided by height in square meters. Fasting blood samples were collected and transferred immediately to a non-heparinized tube for centrifugation. Collected serum was then transferred to pre-labeled plain tubes, stored on ice, and delivered to the Biomarkers Research Program (BRP) in King Saud University, Riyadh, KSA, for immediate storage at −20 °C.

### Sample analyses

Fasting glucose, lipid profile, calcium, and phosphorous were measured using a chemical analyzer (Konelab, Espoo, Finland). Serum 25(OH)D was measured with a Roche Elecsys modular analytics Cobas e411 using an electrochemiluminescence immunoassay (Roche Diagnostics, GmbH, Mannheim, Germany) and commercially available IDS kits (IDS Ltd, Boldon Colliery, Tyne & Wear, UK). Variation for the 25(OH)D ELISA were 5.3 % and 4.6 %, respectively, with 100 % cross-reactivity to 25(OH)D3 and 75 % cross-reactivity to 25(OH)D2. It should be noted that the BRP laboratory is a participating entity in the Vitamin D External Quality Assessment Scheme (DEQAS), and Quality Assurance (QA) standards are maintained by ISO 9000 and 17025. The QA department audits the BRP laboratory at regular intervals. Serum levels (in mmol/L) of glucose <6.1, total cholesterol <5.0, triglycerides <1.7 and calcium 2.25-2.75 were considered normal/desirable.

### Data analyses

Data were analyzed using SPSS (version 16.5 Chicago, IL, USA). Mean and standard deviations were used to represent the data for the Gaussian variables, while median and interquartile range were used to report non-normal variables. Furthermore, frequencies and percentages (%) were reported for the categorical data. Normality assumption was assessed using the Kolmogorov-Smirnov test. Pearson correlation coefficients were calculated for both Gaussian and log-transformed non-Gaussian variables. Multinomial logistic regression was conducted to assess the relationship between select variables and vitamin D status. *P*-value <0.05 was considered statistically significant.

## Results

### Prevalence of vitamin D deficiency

515 normal, healthy women in their 1^st^ trimester of pregnancy were recruited in this prospective study aiming to determine the effect of vitamin D status on the development or not of GDM, during the progression of pregnancy. Blood samples were obtained from these women at the 11.20 (2.75^th^) week of pregnancy and were analyzed for serum 25(OH)D and various other biochemical parameters. Results from the analysis of anthropometric and biochemical parameters of these women are shown in Table 1. For the whole study population (*n* = 515), the means (SD) (in mmol/L) of serum glucose [4.79 (0.76)], total cholesterol [4.70 (1.03)], triglycerides [1.37 (0.59)], calcium [2.27 (0.17)] and corrected calcium [2.24 (0.16)] were within the normal/desirable ranges.

**Table 1:** Anthropometric and clinical characteristics and their association with log serum vitamin D level in pregnant women

Parameters	Mean ± SD	Log Vitamin D Correlation Coefficient (R)	*p*-value
N	515		
Age (years)	28.71 (6.07)	0.100*	0.02
BMI (kg/m^2^)	28.47 (6.22)	−0.002	0.96
Age of menarche (years)	12.95 (1.63)	0.047	0.60
Age at 1^st^ pregnancy (years)	23.57 (4.49)	0.076	0.42
Gestation week	11.20 (2.75)	−0.065	0.30
Waist (cm)	87.54 (12.23)	−0.021	0.82
Hips (cm)	108.01 (12.34)	0.092	0.31
Waist-hip ratio	0.81 (0.07)	−0.124	0.17
Systolic	110.03 (11.56)	0.083	0.11
Diastolic	64.81 (8.72)	0.021	0.68
Gravida#	2.00 (3.00)	0.027	0.61
Parity#	1.00 (2.00)	0.008	0.90
Ca (mmol/L)	2.27 (0.17)	−0.107*	0.02
Total cholesterol (mmol/L)	4.70 (1.03)	0.172**	0.01
Glucose (mmol/L)	4.79 (0.76)	0.090	0.08
Phosphate-inorganic (mmol/L)	1.13 (0.25)	0.090	0.88
HDL-Cholesterol (mmol/L)	1.14 (0.25)	0.017	0.81
Triglycerides (mmol/L)#	1.24 (0.78)	0.184**	0.00
Corrected Ca (mmol/L)	2.24 (0.16)	0.141*	0.03
Vitamin D (nmol/L)#	19.13 (15.08)	--------	--------
Vitamin D - deficient	350 (68 %)	--------	--------
Vitamin D - insufficient	135 (26.2 %)	--------	--------
Vitamin D - sufficient	18 (3.5 %)	--------	--------
Vitamin D - desirable	12 (2.3 %)	--------	--------

Note: Column1: data presented as mean ± standard deviation; # denotes non-Gaussian and data is presented as median (IQR); categorical data presented as frequency and percentages; *p*-value significant at <0.05. Column 2: data presented as coefficient (R);* *p* < 0.05; ** *p* < 0.01

Thirty two (or 6.4 %) of the recruited women had fasting blood glucose level in excess of 6.1 mmol/L. This sub-group of subjects had a mean vitamin D level of 24.42 ± 15.4 nmol/L which was not different from the mean of the whole study group (data not shown). However, because of the small number of women with abnormal levels of glucose and due to the prevalence of vitamin D deficiency in the vast majority of subjects, additional analyses were performed on data from the whole group of recruits. “Hypercholesterolemia” (total cholesterol >5.0 mmol/L) and “hypertriglyceridemia” (triglycerides >1.7 mmol/L) were detected among 32.9 % and 25.8 % of the subjects, respectively.

To gain a better understanding of the relationship between vitamin D and obesity, age, gestational age and parity, multinomial logistic regression analysis was performed for the select variables with vitamin D status (insufficient and deficient) as dependent variable. Results are shown in Table 2. None of the above variables showed any significant association with vitamin D status. However, among the hypertriglyceridemic and hypercholesterolemic women, the latter was significantly associated with insufficient vitamin D status [OR: 13.61 (95 % CI 1.10-168.28)].

**Table 2:** Multinomial logistic regression analysis of vitamin D status versus select parameters

Parameters	Sufficient & Desired Vitamin D (≥50 nmol/L)	Insufficient (between 25–50 nmol/L)		Deficient (<25 nmol/L)	
		OR (95 % CI)	*p*-value	OR (95 % CI)	*p*-value
Hypertriglyceridemia (>1.7 mmol/L)	1.0	0.30 (0.04-1.97)	0.209	0.49 (0.08-2.94)	0.437
Hypercholesterolemia (>5.0 mmol/L	1.0	13.61 (1.10-168.28)	0.042	10.14 (0.86-118.90)	0.065
Obesity (>30 kg/m^2^)	1.0	0.93 (0.15-5.69)	0.938	0.77 (0.14-4.31)	0.762
Gestational Age (≤12 weeks)	1.0	0.23 (0.03-1.53)	0.128	0.24 (0.04-1.49)	0.127
Age (>40 years)	1.0	0.19 (0.01-2.54)	0.210	0.29 (0.03-2.85)	0.289
Multipara (>1)	1.0	0.51 (0.05-5.67)	0.586	0.58 (0.06-6.12)	0.653

Note: *p*-value significant at <0.05; *p*-value significant at <0.01

Based on the previously described clinical categories of vitamin D status [15–18], the subjects were classified into four groups as following: deficient (<25 nmol/L), insufficient (25–50 nmol/L); sufficient (50–75 nmol/L) and desirable (75 < nmol/L). Results indicated that 68, 26.2, 3.5 and 2.3% of the women had deficient, insufficient, sufficient, and desirable levels of vitamin D, respectively (Table 1). The overall study population had a mean serum vitamin D level of 23.36 (15.74) nmol/L.

### Serum vitamin D correlational analysis

Correlation coefficients of various anthropometric and biochemical parameters with log serum vitamin D values, determined as mentioned in Methods, are shown in Table 1. Significant positive associations were detected for serum levels of total cholesterol (R = 0.172; *p* = 0.01), triglycerides (R = 0.184; *p* < 0.01) and corrected calcium (R = 0.141; *p* = 0.03). Besides these, significant associations were also found between serum vitamin D and age in years (R = 0.100; *p* = 0.02). A negative correlation, which however was not statistically significant, was detected for uncorrected calcium (R = −0.107). No additional correlations were found. Furthermore, multiple regression analyses performed using log serum vitamin D status against all metabolic parameters included in the study did not show any significant associations (results not shown).

**Fig. 1:**
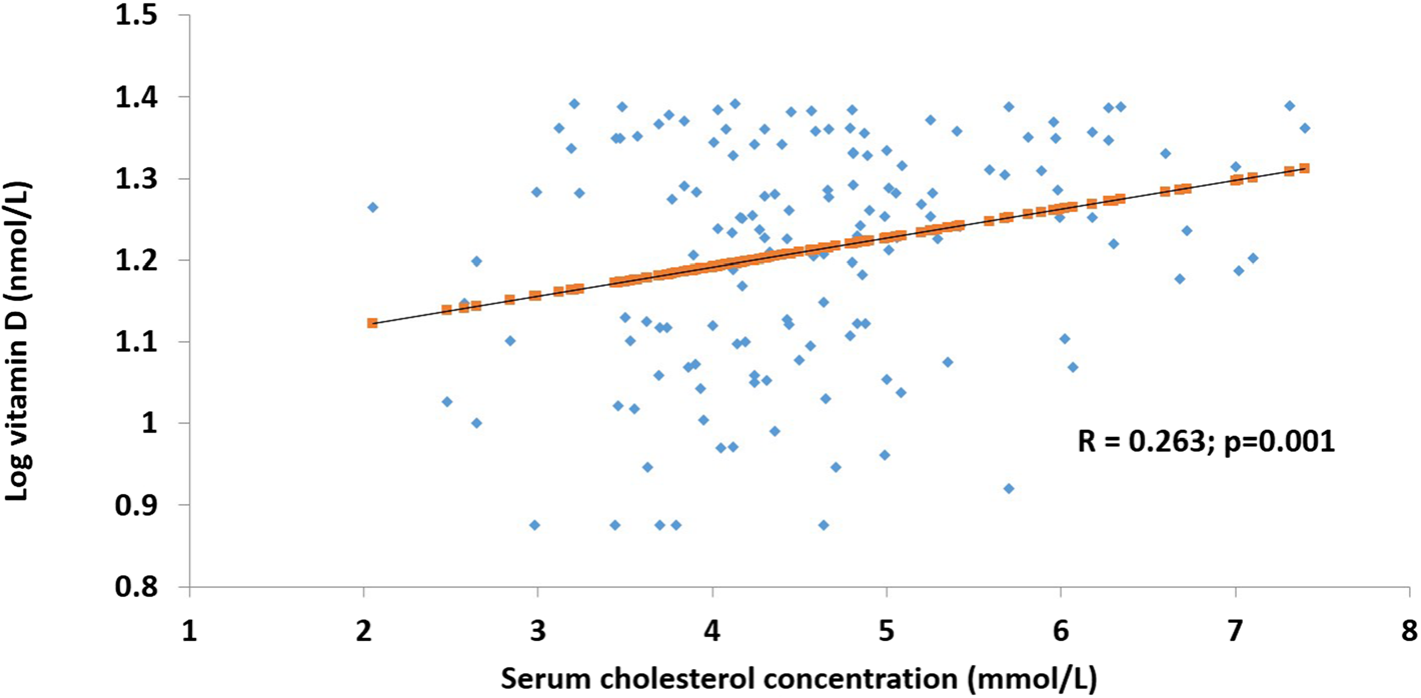
Association between serum log vitamin D and cholesterol concentrations in vitamin D deficient women. In the vitamin D deficient sub-group of women [25(OH)D <25 nmol/L; *n* = 350], the relation between serum levels of log 25(OH)D/vitamin D and total cholesterol was determined by Pearson correlation coefficient analysis. Results are presented as a scatter plot

**Fig. 2:**
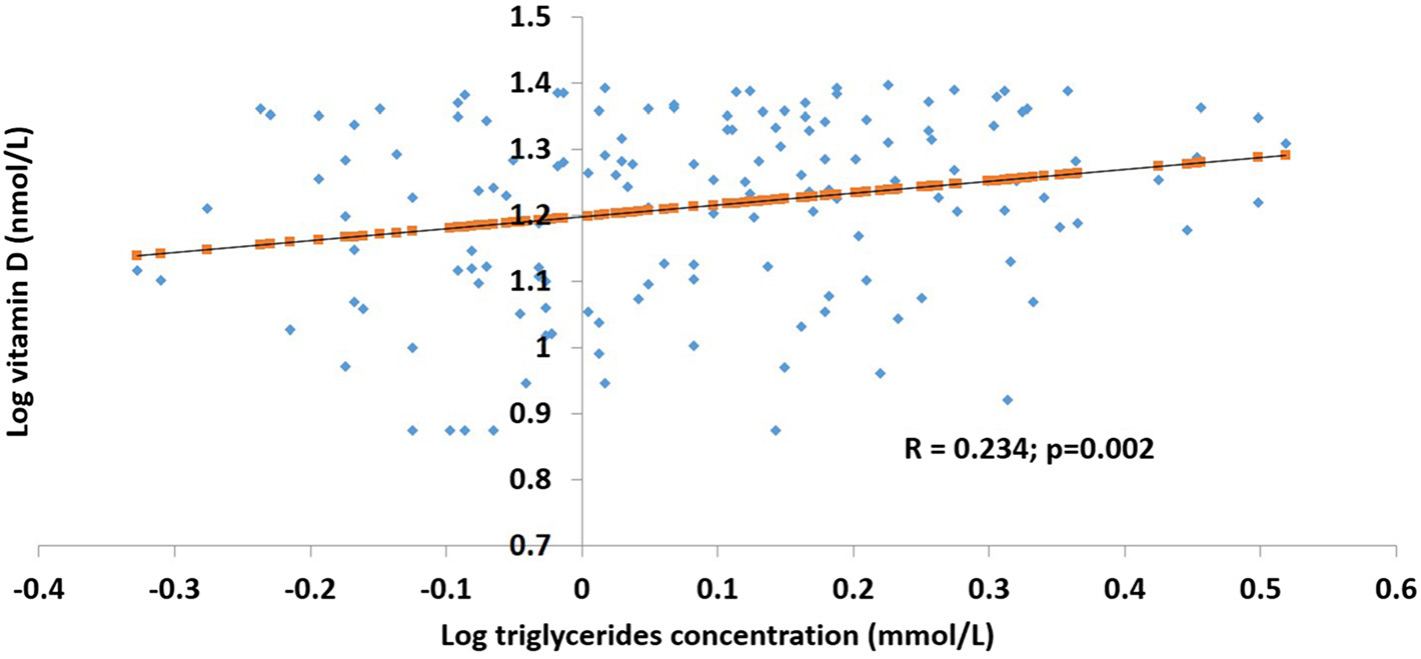
Association between serum log vitamin D and triglyceride concentration in vitamin D deficient women. In the vitamin D deficient sub-group of women [25(OH)D <25 nmol/L; *n* = 350], the relation between serum levels of log 25(OH)D/vitamin D and total triglycerides was determined by Pearson correlation coefficient analysis. Results are presented as a scatter plot

In the vitamin D deficient sub-group of women [25(OH)D <25 nmol/L; *n* = 350], log vitamin D values were positively correlated with serum total cholesterol (R = 0.263; *p* = 0.001) (Fig. 1) and triglycerides (R = 0.234; *p* = 0.002) (Fig. 2).

The mean serum vitamin D levels in the sub-groups of hypertriglyceridemic and hypercholesterolemic subjects were 31.41 (17.29) [control: 26.41 (17.29)] and 30.59 (18.38) [control: 26.82 (17.97)] nmol/L, respectively, and were significantly different (*p* = 0.013 and *p* = 0.021, respectively, from respective controls.

## Discussion

In this study, we assessed the serum vitamin D status of 515 normal, healthy Saudi women in their first trimester of pregnancy, and analyzed its association with several GDM-related anthropometric and serum biochemical markers. Vitamin D deficiency was highly prevalent, with only 3.5 % of the women having sufficient and an additional 2.5 % of the women having desirable levels of vitamin D, while the rest (94.2 %) were either insufficient or deficient. Thirty two of these women had abnormally high level of fasting glucose. Hypercholesterolemia and hypertriglyceridemia were detected among 25.8 % and 32.9 % of the subjects, respectively. Serum vitamin D level was positively correlated to total cholesterol, triglycerides and corrected calcium in our study subjects, while the mean serum levels of these same parameters were within their normal ranges for the total population. Vitamin D level was even more strongly correlated to total cholesterol and triglycerides in the vitamin D-deficient sub-group of women. However, regression analysis indicated a modest risk of hypercholesterolemia among women with insufficient vitamin D. We speculate that the unexpected positive correlations between vitamin D and lipids may be the outcome of a combination of deficient vitamin D status with the high metabolic demands of pregnancy.

Recent research at the cellular level indicates pleiotropic roles for vitamin D, while clinical trials involving high doses have not been associated with toxicity. Hence, intense research efforts are being made to determine the normal/deficiency levels with a view to normalize vitamin D status, via dietary supplementation, in persons with chronic diseases and in children, pregnant women and adults for possible prevention of the same. East African populations with traditional lifestyles having lifelong, year-round exposure to tropical sunlight have a mean serum vitamin D concentration of 115 nmol/L, independently of age, sex or BMI [19]. The pregnant adults of these populations had higher serum vitamin D than their non-pregnant counterparts. Most investigators agree that serum vitamin D should be higher than 50 nmol/L, but some recommend even higher serum levels. Various factors, such as race and geographical location, affect vitamin D levels and risk groups for vitamin D deficiency include pregnant women, children, older persons, the institutionalized and non-western immigrants. Vitamin D deficiency in the Middle-East adults is highly prevalent [20]. The Institute of Medicine (IOM) recently revised the recommended dietary allowances (RDA) for vitamin D (above 50 nmol/L) to sustain normal bone density, calcium absorption, and to minimize risk of osteomalacia and rickets. Results from many clinical trials that show benefits and/or no adversity with doses of vitamin D that raise serum vitamin D to levels beyond 75 nmol/L support the desirability of having serum vitamin D levels greater than 75 nmol/L [18].

Several recent studies have reported widespread prevalence of hypovitaminosis D among adolescent girls and pregnant and post-natal women in Saudi Arabia and around the world [21, 22]. Even though hypovitaminosis D (<50 nmoles/L) was highly prevalent (96.3 % of the subjects), inter-trimester variations were not observed in a cross-sectional study involving 541 women in India [23]. Vitamin D deficiency in an earlier study on healthy Saudi pre-and postmenopausal women was related to obesity, poor exposure to sunlight, and poor dietary vitamin D supplementation [24]. However, in another study, a cohort of 139 healthy young Saudi Arabian blood donors (87 males and 52 females) had vitamin D deficiency, despite the fact that >65 % of the participants had adequate exposure to sunlight, while > 90 % reported adequate intake of dairy products [25].

6.4 % of the pregnant women of this study had higher than expected serum glucose level, indicating several candidates for possible gestational diabetes. According to a 2014 analysis by the US Centers for Disease Control and Prevention, the prevalence of GDM was as high as 9.2 %; further, while the severity of GDM increased with the progression of pregnancy, with the whole-body insulin sensitivity reduced 45 % to 70 % below non-pregnant values during the third trimester of pregnancy [26]. The oral glucose tolerance test (OGTT) planned among these women in the latter part of their pregnancy is expected to provide us a better understanding of the relation between vitamin D and GDM.

Free/ionic calcium in all the subjects or in the vitamin D-deficient sub-group of pregnant women was significantly related to vitamin D. The mechanistic basis of ionic calcium homeostasis is still not completely established. However, the roles of the important individual players of calcium homeostasis aid in hypothesizing that serum levels of free/ionized calcium may be reciprocally regulated by parathyroid hormone (PTH) and 1, 25-dihydroxyvitamin D, with the former one serving to increase serum level of calcium and the latter to suppress it. PTH causes net bone loss (resorption) and increases blood calcium levels by stimulating osteoclasts. High levels of vitamin D have been shown to inhibit PTH synthesis *in vitro* and *in vivo* [27, 28]. Vitamin D deficiency is implicated in reduced serum albumin concentrations [29]. Hence, serum ionic/free calcium, which is non-albumin bound calcium, is expected to be higher in vitamin D deficiency conditions.

Plasma vitamin D levels are elevated in normal early pregnancy and continue to increase throughout pregnancy, while they remain elevated postpartum in lactating women [30]. The elevated levels probably represent a physiologic response to increased calcium requirements. Calcitonin is a major positive regulator for the expression of renal 25-hydroxyvitamin D3-1-alpha-hydroxylase gene in normocalcemic rats. Calcitonin selectively stimulates 25-hydroxyvitamin D3-1-alpha-hydroxylase in the proximal convoluted tubule of rat kidney [31]. Plasma-calcitonin levels were significantly higher throughout normal pregnancy and lactation than in normal non-pregnant women [32]. Thus, more calcitonin circulates at times of physiologically increased calcium needs.

The increases in calcitonin and vitamin D may be important in the transfer of maternal calcium to the fetus and in the prevention and recovery of maternal bone loss. Pregnancy and lactation place unique metabolic demands. Increased insulin, as well as higher level of glucose, are observed in gestational diabetes mellitus, possibly as a result of the progressively increasing insulin resistance in pregnancy. Similarly, hypercalcemia and increased vitamin D may occur together in pregnancy.

Several studies have shown an “atherogenic” metabolic bias during normal pregnancy, whose intensity increased with progression of pregnancy. Increases in the levels of triglycerides and unchanged HDL-cholesterol levels were associated with GDM [11, 33]. Exaggerated hypertriglyceridemia, particularly in the VLDL and HDL fractions, was a feature of GDM [34]. In VLDL, core lipids (TG + cholesterol) increased over normal gestation and were greater in GDM then women with no GDM.

Although inconclusive, vitamin D is generally considered anti-atherogenic, as several observational studies and recent meta-analyses in humans showed an inverse correlation between circulating vitamin D and poor cardiovascular outcomes [35–37]. Besides, vitamin D activates cholesterol efflux from macrophages, a critical component in atherosclerotic plaque regression [38], inhibits cholesterol synthesis by inhibiting HMG CoA reductase [39] and suppresses lipolysis in adipocytes stimulated by the β-adrenergic receptor pathway through increasing intracellular calcium levels [40, 41].

Despite all these purported anti-atherogenic actions of vitamin D, our results indicate significant positive correlations between serum 25(OH)D levels and the atherogenic factors total cholesterol and triglycerides. A plausible explanation for the positive correlation in the pregnant woman may be a normal response to pregnancy, as tissues such as liver, muscle and fat may become more responsive to placental hormones; this is supported by the finding that progesterone increased cholesterol synthesis, which was inhibited by various statins [42]. Further, the hyperglycemic and hyper-insulinemic and condition of GDM may facilitate synthesis of cholesterol by their direct influence on liver and adipose tissues [43]. Serum triglycerides in pregnant women are increased 1.5 to 2-fold above non-pregnant concentrations by the third trimester and remain high around the clock [44], and this is attributed mainly to a combination hyperinsulinemia promoting triglyceride synthesis in the liver, increased food intake resulting in increased appearance of chylomicrons from the gut and reduced activity of lipoprotein lipase in adipose tissue, resulting in decreased clearance of triglycerides from the circulation [45]. Thus, besides influencing carbohydrate metabolism, insulin and placental hormones, such as human placental lactogen, secreted during the course of pregnancy stimulate, either directly or indirectly some of the adaptations of maternal lipid metabolism. The major tissues affected by human placental lactogen are the liver, skeletal and heart muscles and adipose tissues.

Also, a modest positive relation was found between insufficient vitamin D status and hypercholesterolemia through regression analysis. The study subjects consisted of women in their 1^st^ trimester of pregnancy; future follow-up studies on these same women during the successive trimesters were planned and these might provide better clues in understanding the relation between vitamin D deficiency and pregnancy-induced atherogenic lipid metabolic markers. A quantitative relation between serum vitamin D and atherogenic markers during pregnancy is expected to help reveal the role of vitamin D in pregnant and perinatal women and provide useful clues regarding the optimal level of vitamin D supplementation in pregnancy.

## Conclusions

Vitamin D is an endogenous hormone with pleiotropic roles in normal physiology. Under normal conditions, vitamin D exerts anti-atherogenic, anti-diabetic, anti-osteoporotic activities. In this study, women in the first trimester of pregnancy had high prevalence of vitamin D deficiency; surprisingly, however, serum vitamin D in these subjects correlated positively with serum levels of triglycerides, cholesterol and corrected calcium. We believe that this abnormal association may represent adaptation to the high metabolic demands of pregnancy and future studies during later trimesters planned for these women are expected to provide better explanation.
